# Nutrient pathways and their susceptibility to past and future change in the Eurasian Arctic Ocean

**DOI:** 10.1007/s13280-021-01673-0

**Published:** 2021-12-16

**Authors:** Robyn E. Tuerena, Claire Mahaffey, Sian F. Henley, Camille de la Vega, Louisa Norman, Tim Brand, Tina Sanders, Margot Debyser, Kirstin Dähnke, Judith Braun, Christian März

**Affiliations:** 1 Scottish Association for Marine Science, Oban, PA37 1QA UK; 2 Department of Earth, Ocean and Ecological Sciences, School of Environmental Sciences, University of Liverpool, 4 Brownlow Street, Liverpool, L69 3GP Merseyside UK; 3 School of GeoSciences, University of Edinburgh, James Hutton Road, Edinburgh, EH9 3FE UK; 4 Institute for Carbon Cycles, Helmholtz-Zentrum Hereon, Max-Planck-Str. 1, 21502 Geesthacht, Germany; 5 School of Earth & Environment, University of Leeds, Leeds, LS2 9JT UK

**Keywords:** Arctic, Climate change, Ecosystems, Nitrate, Nutrients, Phosphate, Productivity

## Abstract

Climate change is altering nutrient cycling within the Arctic Ocean, having knock-on effects to Arctic ecosystems. Primary production in the Arctic is principally nitrogen-limited, particularly in the western Pacific-dominated regions where denitrification exacerbates nitrogen loss. The nutrient status of the eastern Eurasian Arctic remains under debate. In the Barents Sea, primary production has increased by 88% since 1998. To support this rapid increase in productivity, either the standing stock of nutrients has been depleted, or the external nutrient supply has increased. Atlantic water inflow, enhanced mixing, benthic nitrogen cycling, and land–ocean interaction have the potential to alter the nutrient supply through addition, dilution or removal. Here we use new datasets from the Changing Arctic Ocean program alongside historical datasets to assess how nitrate and phosphate concentrations may be changing in response to these processes. We highlight how nutrient dynamics may continue to change, why this is important for regional and international policy-making and suggest relevant research priorities for the future.

**Supplementary Information:**

The online version contains supplementary material available at 10.1007/s13280-021-01673-0.

## Introduction

The Arctic is warming at twice the rate of the global average, causing rapid changes to the marine ecosystem. These changes are having impacts locally, regionally and on a global scale. Approximately 50% of the Arctic Ocean is made up of productive shelves supporting large fisheries and diverse habitats. These shelf seas not only play a key role in local and global biogeochemical cycles and climate but are also economically important regions. Arctic primary production has increased by > 50% in the last two decades (Lewis et al. 2020); these trends were initially driven by increased light availability, but enhanced chlorophyll-a concentrations between 2009 and 2018 suggest there has also been an increase in nutrient availability to sustain enhanced growth. Whether this trend continues will depend on whether there is a sustained nutrient supply to surface waters (Arrigo and van Dijken 2015; Lewis et al. 2020).

Currently, nitrogen (N) is considered to be the main nutrient limiting primary productivity in the Arctic Ocean (Mills et al. 2018; Ko et al. 2020), but this is mostly based on studies in Pacific-influenced waters of the western Arctic. The eastern Arctic is less N-limited and nitrate, silicate and iron all appear to play a role in regulating primary production (Krisch et al. 2020). There has been an 88% increase in primary production in the Barents Sea over the past two decades at a rate of 3.73 Tg C year^−1^, which is greater than the average change for the whole Arctic (57%) and faster than other regions (~ 1 Tg C year^−1^ or less (Lewis et al. 2020). For these changes to continue in the future, the standing stock of nutrients in the Barents Sea must decrease as primary production continues to increase, or there must be an increase in the nutrient supply to this region over time.

**Fig. 1:**
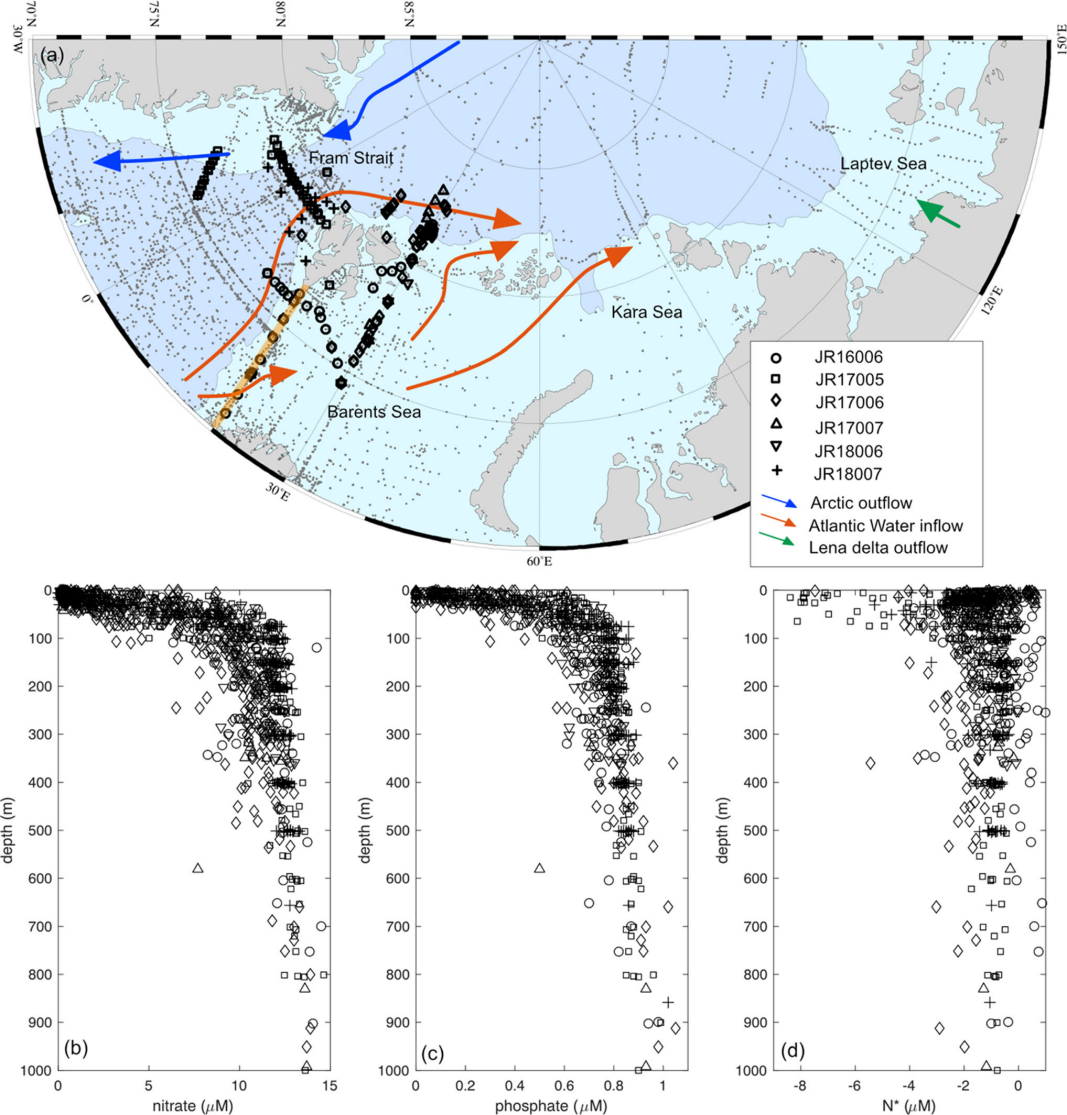
**a** Map of the Eurasian Arctic highlighting the Changing Arctic Ocean cruises (JR16006, JR17005, JR17006, JR17007, JR18006 and JR18007), the Codispoti et al., (2013) dataset (grey dots), the Atlantic Water inflow (orange arrows), the Arctic Water outflow (blue arrows) and the Lena Delta (green arrow). The orange line shows the M/S Norbjørn transect across the Barents Sea Opening. **b** nitrate, **c** phosphate and **d** N* profiles from the Changing Arctic Ocean cruises over three consecutive years (2017, 2018 and 2019) covering the Fram Strait and Barents Sea

Atlantic Water (AW) is supplied to the Eurasian Arctic via the Fram Strait and the Barents Sea Opening (BSO) (Fig. 1a). Atlantic Water supplies nutrients to the Eurasian shelves with nitrate and phosphate concentrations close to the Redfield ratio (15–16N:1P), and low concentrations of silicate, which can limit the extent of diatom growth (Hatun et al. 2017). As warm and saline AW is transported across the Barents Sea, it is modified by atmospheric cooling and mixed with cold, fresh Arctic Water (ArW) and the Norwegian Coastal Current (Rudels et al. 1996). The full water column is experiencing increased ocean heat transport from the Atlantic (Arthun et al. 2012; Onarheim et al. 2015), amplified atmospheric warming and increases in salinity (Barton et al. 2018; Lind et al. 2018). On the eastern side of Fram Strait, AW is transported northward within the West Spitsbergen Current (WSC) and enters the Arctic Ocean north of Svalbard (Fig. 1). In the upper layers of the WSC, the AW has been warming since the mid-1990s at a rate of 0.06 °C year^−1^ and also increasing in salinity (Beszczynska-Moller et al. 2012; Polyakov et al. 2017; Tsubouchi et al. 2021).

In the Eurasian Arctic, the areal extent of AW is increasing (Oziel et al. 2018, 2020) and the northern Barents Sea is now transitioning to a regime of weakened stratification and enhanced vertical mixing (Lind et al. 2018), where winter and summer sea ice cover is rapidly declining (Arthun et al. 2012; Onarheim and Arthun 2017). The weakened stratification of AW compared to the salinity-stratified ArW is leading to enhanced fluxes of nutrients to the surface ocean (Randelhoff et al. 2018, 2019; Tuerena et al. 2021a), which may be sustaining phytoplankton blooms for a longer period (Henley et al. 2020). Increased nutrient supply has also been observed with sea ice retreat through strengthened upwelling (Tremblay and Gagnon 2009) and increased storminess (Yang et al. 2004).

In contrast to these findings, other studies have identified processes that may reduce nutrient availability in the Eurasian Arctic. The AW inflow is a mixture of nutrient-rich North Atlantic subpolar and nutrient-poor subtropical water advected into the Norwegian Sea. Over the last two decades, silicate concentrations have decreased in AW, driven by shallower winter mixing in the subpolar gyre, coupled with weakening and westward retraction of the gyre which has increased the proportion of subtropical water entering the Norwegian Sea (Hatun et al. 2017). Further to this, freshwater dilution and warming (McLaughlin and Carmack 2010; Nummelin et al. 2015) may reduce nutrient availability in the surface ocean.

Through the UK-led Changing Arctic Ocean programme, the biogeochemistry of the Arctic Ocean has been investigated through international research efforts and multi-year cruise campaigns in the Eurasian Arctic (Fig. 1). Here we summarise some of the findings from this programme describing the nutrient biogeochemistry of the Eurasian Arctic, its sensitivity to future change and the past and future implications for primary production (Table 1). We explore seasonal and long-term trends in nutrient availability and the role that changes to water mass circulation may have in determining primary production. We focus particularly on the cycling and stoichiometry (relative ratios) of nitrate and phosphate, the key macronutrients necessary for all phytoplankton, through seasonal cycling in the water column, interactions with sea ice, benthic pelagic coupling and riverine inputs to the Eurasian Arctic alongside long-term trends in nutrients.

**Table 1:** Statistical parameters of the temporal linear models for nitrate, phosphate and N* per year for each depth zone

Depth	Model	Response variable	Explanatory variable	*n*	Intercept (± SD)	*p* value	Slope (± SD)	*p* value	*R*^2^ (%)	*F* value (DF)
< 25 m	Linear	Nitrate	Year	254	192.62 ± 56.16	< 0.01	− 0.094 ± 0.028	< 0.01	3.9	11.28 (252)
< 25 m	Linear	Phosphate	Year	527	9.29 ± 2.42	< 0.01	− 0.004 ± 0.001	< 0.01	2.3	13.54 (525)
**< **25 m	Linear	N*	Year	237	− 64.62 ± 23.68	< 0.01	0.032 ± 0.011	< 0.01	2.5	7.12 (235)
**< **100 m	Linear	Nitrate	Year	298	188.84 ± 33.22	< 0.01	− 0.090 ± 0.017	< 0.01	8.8	29.74 (296)
**< **100 m	Linear	Phosphate	Year	616	10.48 ± 1.62	< 0.01	− 0.005 ± 0.001	< 0.01	5.6	37.0 (614)
**< **100 m	Linear	N*	Year	268	− 44.50 ± 18.37	0.016	0.022 ± 0.009	0.020	1.7	5.5 (266)
200–300 m	Linear	Nitrate	Year	224	100.84 ± 13.77	< 0.01	− 0.044 ± 0.007	< 0.01	15.3	41.31 (222)
200–300 m	Linear	Phosphate	Year	224	13.04 ± 1.92	< 0.01	− 0.006 ± 0.001	< 0.01	15.0	40.43 (222)
200–300 m	Linear	N*	Year	224	− 37.90 ± 10.75	< 0.01	0.019 ± 0.005	< 0.01	4.7	11.91 (222)

*DF* degree of freedom, *n* number of samples, *SD* standard deviation, *nitrate* nitrate + nitrite

## Methods

Sampling was conducted onboard the RRS James Clark Ross during six research expeditions to the Barents Sea and Fram Strait from 2017 to 2019. Dissolved inorganic nutrient samples (nitrate + nitrite, nitrite, silicate and phosphate) were collected from the CTD Niskin bottles, filtered and frozen until analysis at the University of Liverpool or measured onboard. Additional sampling was conducted in collaboration with the Norsk Institutt for Vannforskning (NIVA, Oslo) during transits made by the general cargo vessel M/S Norbjørn between Tromsø, Norway and Longyearbyen, Svalbard. The M/S Norbjørn is a ‘ship of opportunity’ onto which NIVA has fitted a FerryBox system that measures physical parameters at approximately 4 m depth. During each 4-day transit in March, June, August and November 2018 and 2019, surface seawater samples were collected from 15 stations at pre-determined latitudes (Fig. 1). Seawater was filtered through combusted GF/F filters and aliquots of the filtrate were frozen until analysis.

Onshore nutrient measurements were conducted at the University of Liverpool using a Bran and Luebbe, QuAAtro 5-channel continuous flow analyzer. Manufacturer-recommended methods for detection in seawater were used: Phosphate Q-064–05 Rev. 2, Nitrate + Nitrite using a Cd coil Q-068-05 Rev. 2, Nitrite Q-070-05 Rev. 2. Samples were warmed to room temperature prior to analysis. Samples were analysed in triplicate in batch sizes of 20–30 and working standards were freshly made daily. Kanso certified reference material (CRM) for nutrients in seawater (Kanso Co Ltd, Lot CI) were used during every run. CRMs were run in triplicate every 5 samples, including the start and end. Overall, phosphate accuracy and precision were better than 98.7% and 1.5%, respectively, and nitrate + nitrite accuracy and precision were better than 98.2% (mostly > 99%) and 1%, respectively*.* Detection limits were 0.03 µM for nitrate + nitrite, 0.02 µM for phosphate and 0.2 µM for silicate. Herein the measurement of nitrate + nitrite is defined as nitrate.

These measurements were compared to published nutrient datasets from the Eurasian Arctic (Tables S1, S2, S3). To explore decadal trends in nitrate and phosphate, statistical analyses were performed in R version 3.5.1 (R Core Team 2018). To analyse temporal variation in nitrate, phosphate and N* (N* = nitrate − phosphate × 16 (Gruber and Sarmiento 1997)), linear models were fitted with nitrate, phosphate and N* as a function of year for surface (< 25 m), intermediate (< 100 m) and deep (200–300 m) waters separately. We used the Gaussian family with no transformation of the data, assuming that measurement errors were normally distributed. Model fit was checked by residual analyses with visual inspection of quantile–quantile plots, and residuals and standardized residuals versus fitted values plots. *p *Values, R2, *F*-statistics, and degrees of freedom are reported for each model (Table 1).

Inorganic and organic nutrient concentrations were also collected from the Lena Delta and used to describe changes across the Laptev Sea (Sanders et al. 2022). To investigate the role of nitrogen limitation, the semi-conservative tracer N* was calculated from N* = nitrate−phosphate × 16 (Gruber and Sarmiento 1997) (Fig. 1d). The role of organic nutrients was also investigated through the semi-conservative tracer TDN* = TDN–TDP × 16 (TDN = total dissolved nitrogen, TDP = total dissolved phosphorus). We use the same stoichiometry to compare changes in total nutrient concentrations to the average stoichiometry of marine phytoplankton.

Sediment porewater samples were collected from triplicate multi-corer deployments during cruise JR16006 using rhizon syringe filters (pore size 0.15 µm). Vertical sampling resolution was 0.5 cm in the upper 2 cm, 1 cm from 2 to 10 cm and 2 cm below 10 cm depth. Bottom water was also sampled on core recovery. Porewater samples were analysed for concentrations of nitrate + nitrite, nitrite, ammonium, silicate and phosphate using a Lachat Quikchem 8500 flow injection autoanalyser. Samples were diluted by 1/3 with low nutrient seawater from OSIL (Ocean Scientific International Ltd., Batch 25) and analysed against a set of five calibration standards also made up in a low nutrient seawater matrix. Analytical performance was assessed using CRMs (KANSO Co Ltd, Lot CG) and an internal standard made up in low nutrient seawater. Analytical precision was generally better than 2% for nitrate + nitrite, phosphate and ammonium. Detection limits were 0.1 µM for nitrate + nitrite, phosphate and ammonium. Porewater profiles of nutrient concentrations were used to estimate diffusive nutrient fluxes across the sediment–water interface according to Fick’s first law of diffusion (Eq. 1).

1 $$J_{{{\text{sed}}}} = \phi x \, D_{{{\text{sed}}}} x{\text{ d}}C/{\text{d}}x,$$

*J*_sed_ is the sediment–water diffusive flux of each nutrient, *ϕ* is the sediment porosity, *D*_sed_ is the diffusion co-efficient of each nutrient in sediment, and d*C*/d*x* is the nutrient concentration gradient.

## Results

### Seasonal and decadal variability in water column nutrients

**Fig. 2:**
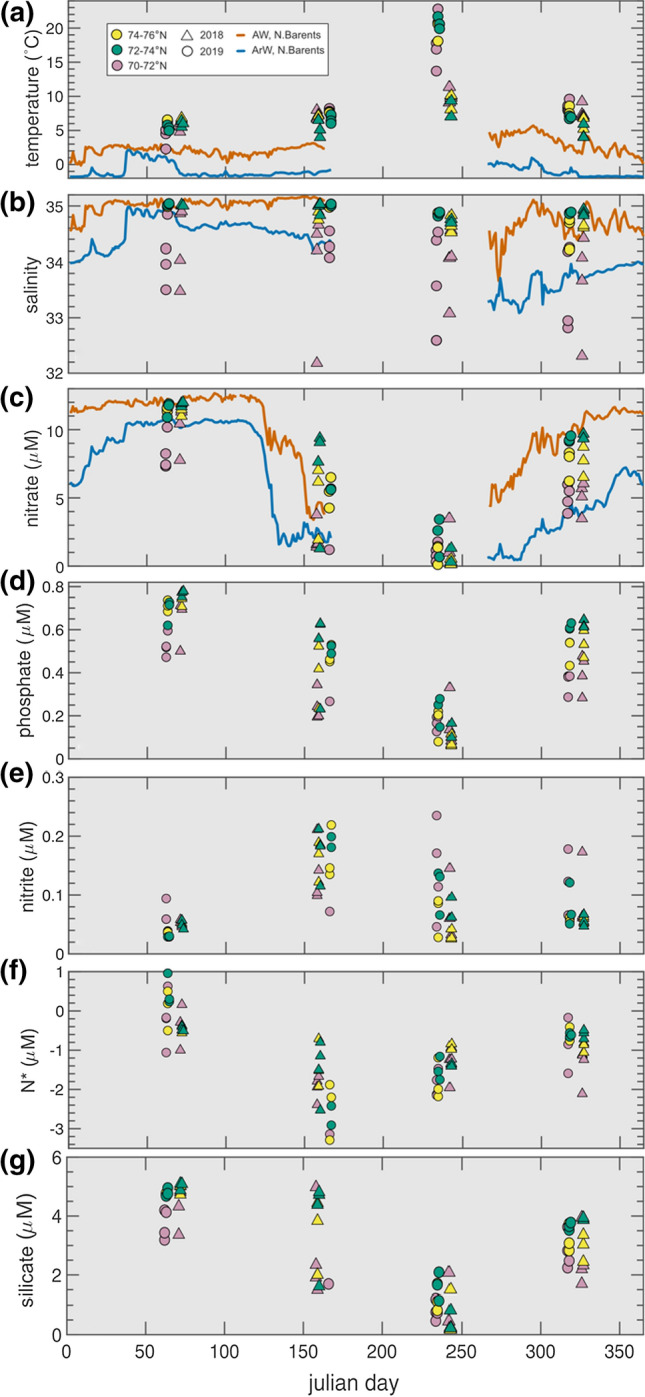
Seasonal and interannual variability in **a** temperature, **b** salinity, **c** nitrate, **d** phosphate, **e** nitrite, **f** N* and **g** silicate across the Barents Sea Opening. Samples were collected from the surface (4 m) during 2018 (triangle) and 2019 (circle) over four months (March, June, August and November). We used the average SST and salinity values between 2010 and 2016 to separate the transect into three regions representing different water masses: the southern coastal region between 70 and 72°N (pink), influenced by terrestrial, freshwater inputs; 72–74°N region, where Atlantic Waters enter the BSO (green); and 74–76°N region that is situated parallel to the polar front that is shaped by the bathymetry and hydrology surrounding Bear Island (yellow). In **a**–**c**, line plots are shown to depict the AW (orange) and ArW (blue) trends from mooring data at 21 m, as described in Henley et al., (2020)

Seasonal datasets can be used to determine the importance of nutrient uptake, limitation and recycling through the onset of summer productivity and the subsequent replenishment of nutrients over winter months. We measured variability in surface macronutrient concentrations across the Barents Sea Opening in four months (March, June, August and November) spanning the years of 2018 and 2019 (Fig. 2). These data were compared to mooring data from the northern Barents Sea, which captured near-surface variability in nitrate concentration, temperature and salinity in AW and ArW (Henley et al. 2020).

Nutrient uptake commenced from late May–June onwards, with initial uptake of nitrate and phosphate exceeding Redfield (16:1) ratios leading to a depletion in N* (− 2.1 ± 0.88 μM) (Fig. 2f). As phytoplankton growth continued through the summer, nitrate and phosphate were depleted in surface waters, with concentrations of 1.0 ± 1.02 μM and 0.15 ± 0.08 μM, respectively, remaining in late August following consumption. Nitrate concentrations were highest in late winter (March), from replenishment over winter months and N* concentrations were restored to Redfield concentrations (− 0.21 ± 0.51 μM), comparable to the AW. Nitrite remained below 0.25 μM throughout all seasons and was lowest in March, suggesting that the intermediate products in N recycling processes, ammonium and nitrite, had been nitrified to nitrate, either in-situ or through winter convection and nitrification (Fig. 2e). The highest nitrite concentrations were during or following the spring/summer blooms, and remained high into November.

**Fig. 3:**
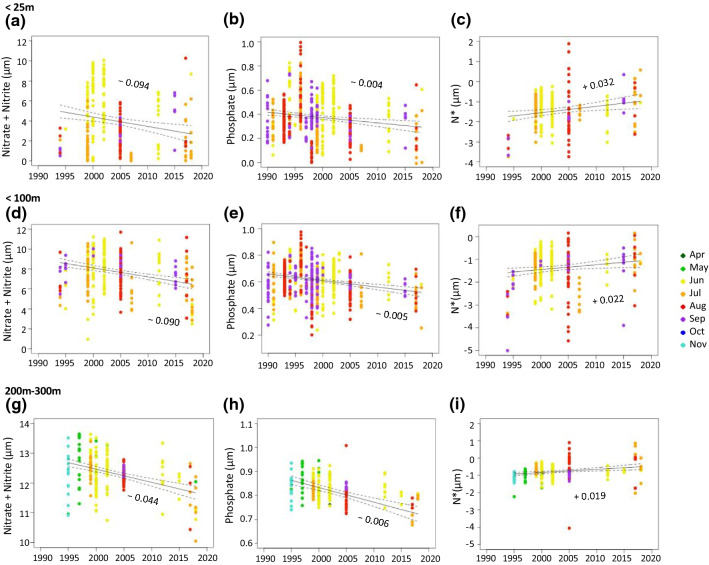
Decadal trends in nutrient concentration. Concentration (µM) of nitrate + nitrite (**a**), phosphate (**b**) and N* (**c**) in surface water (< 25 m), concentration (µm) of nitrate + nitrite (**d**), phosphate (**e**) and N* (**f**) integrated over the upper 100 m, and concentration (µm) of nitrate + nitrite (**g**), phosphate (**h**) and N* (**i**) in deep water (200–300 m) per year

We combined our data from the CAO field programme with historical datasets to assess the decadal trends in nutrient availability in the Barents Sea and the eastern Fram Strait (Fig. 3). We used three approaches: we assessed variability firstly, in surface nutrients in summer (upper 25 m, June–September) (Table S1); secondly, in nutrient concentrations in the upper 100 m (June–September) (Table S2) and thirdly, in deep AW (200–300 m, throughout the year) (Table S3). We used these approaches to determine whether there is an absolute change in the nutrient inventory from the AW source to the Eurasian Arctic, or through increased primary production or a relative change in the water column distribution of nutrients through changes in upper ocean mixing.

Nitrate and phosphate significantly decreased with time at all depths (1994–2019), and N* significant increased with time at all depths (linear models: *p* < 0.05; Table 1; Fig. 1). The concentration of nitrate in the upper 25 m decreased by 0.094 ± 0.028 µM year^−1^ (linear model: *p* < 0.01; Table 1) (Fig. 3a). The concentration of phosphate decreased by 0.004 ± 0.001 (linear model: *p* < 0.01; Table 1) and N* increased by 0.032 ± 0.011 µM year^−1^ (linear model: *p* < 0.01; Table 1) (Fig. 3c). The concentration of nitrate in the upper 100 m decreased by 0.090 ± 0.017 µM year^−1^ (linear model: *p* < 0.01; Table 1) (Fig. 3d). The concentration of phosphate decreased by 0.005 ± 0.001 µM year^−1^ (linear model: *p* < 0.01; Table 1) (Fig. 3e) and N* increased by 0.022 ± 0.009 µM year^−1^ (linear model: *p* = 0.02; Table 1) (Fig. 3f). In the deeper AW, the concentration of nitrate decreased by 0.044 ± 0.007 µM year^−1^ (linear model: *p* < 0.01; Table 1) (Fig. 3g). The concentration of phosphate decreased by 0.006 ± 0.001 µM year^−1^ (linear model: *p* < 0.01; Table 1) and N* increased by 0.019 ± 0.005 µM year^−1^ (linear model: *p* < 0.01; Table 1, Fig. 3i).

### Benthic and riverine nutrient cycling

**Fig. 4:**
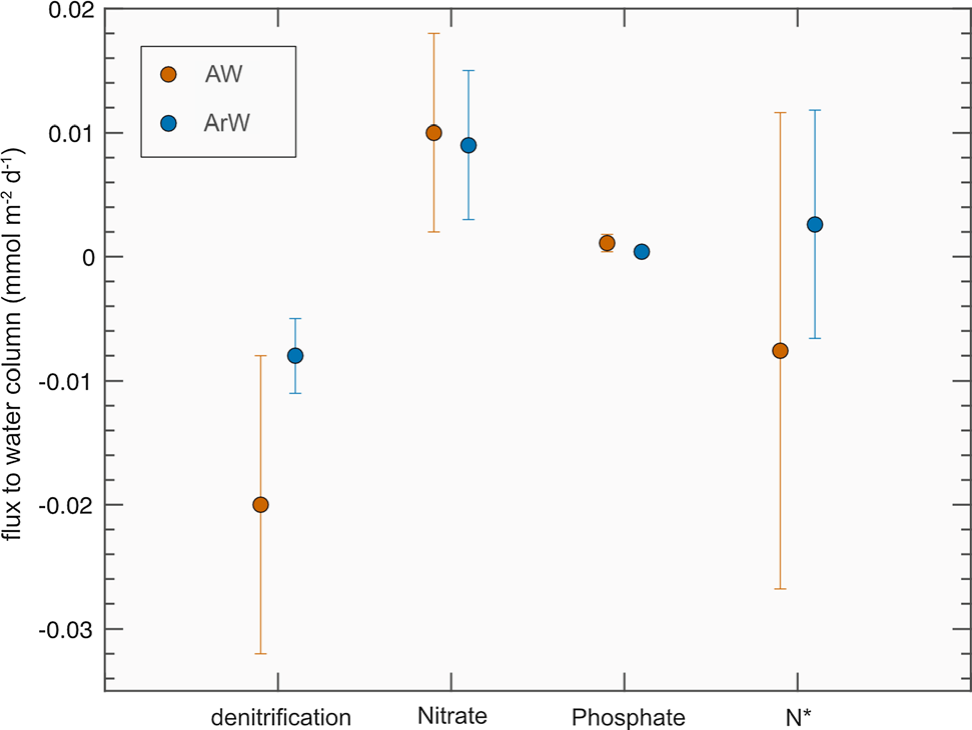
Benthic flux estimates from Atlantic Water stations (orange) and Arctic Water (blue) of N loss by denitrification, nitrate flux to the water column, phosphate flux to the water column and N* flux to the water column from the Barents Sea in 2017

We used further data from the CAO programme to investigate how sediment and riverine processes may alter nutrient cycling over the Eurasian shelves (Fig. 1a). The role of benthic denitrification was explored using nutrient porewater data from AW and ArW influenced sites in the Barents Sea (Figs. 1 and 4). Although only representing a snapshot in time and space, these porewater flux estimates give an indication of the processes that may influence nitrate and phosphate cycling in the benthic environment and exchange with overlying waters.

Our flux estimates showed that denitrification occurs in shallow sediments at our sampling sites, but accounted for only a small proportion of biological cycling and shelf nutrient budgets (Fig. 4). The return flux of nitrate to bottom waters occurred when nitrification rates exceeded denitrification rates, but this measured return flux was also small. There was no significant difference between total nitrate and phosphate fluxes, or denitrification rates, at AW-dominated stations compared to ArW-dominated stations.

**Fig. 5:**
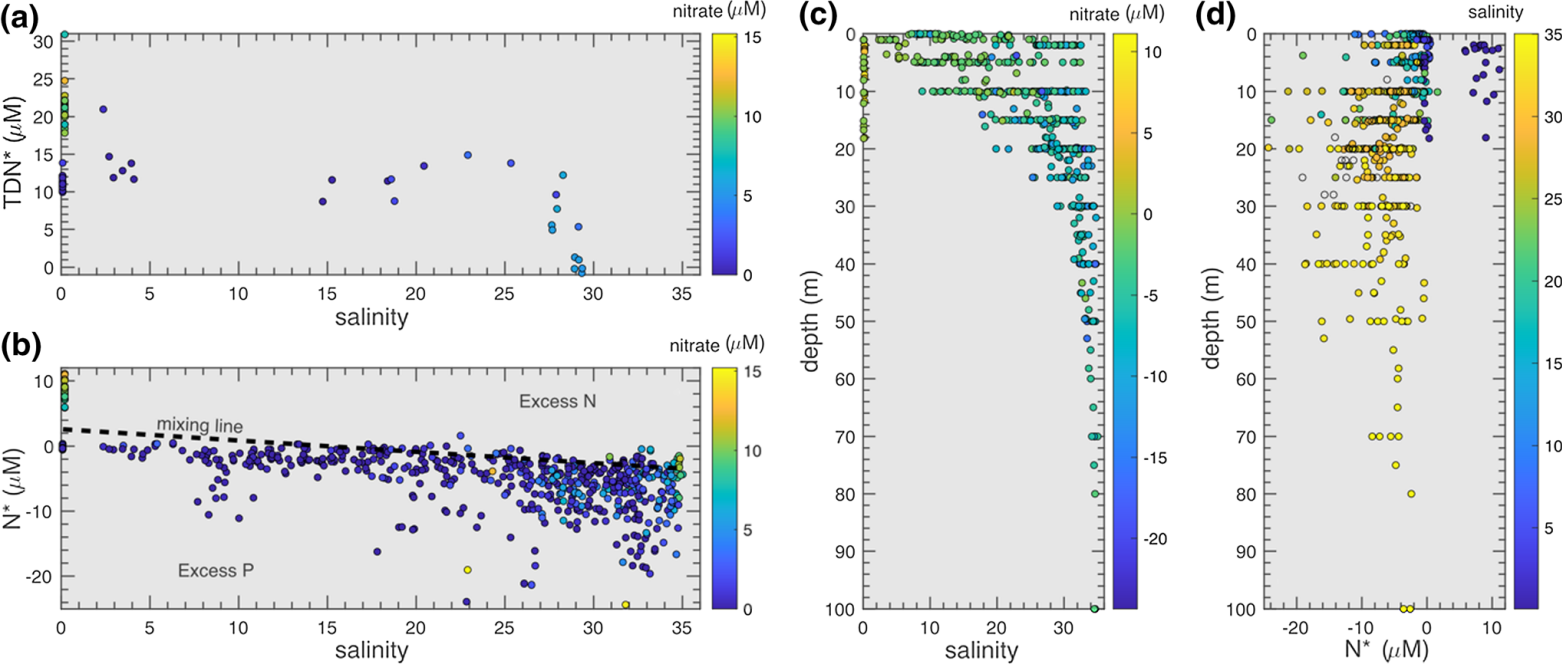
Variability in **a** TDN* and **b** N* with changing salinity through the Lena Delta and into the Laptev Sea with nitrate plotted in colour. Variability in **c** salinity and **d** N* in the upper 100 m of the Lena Delta and Laptev Sea with salinity plotted in colour. Data from the Lena Delta are from Sanders et al. (2022). A mixing line is added to **b** between the marine (N* = -3.3 μM, salinity = 34.8) and riverine endmembers (N* = 2.2 μM, salinity = 0)

The Eurasian shelves are supplied with freshwater and nutrients from large Siberian rivers, we have utilised new data from the Lena delta to capture the changing biogeochemistry from freshwater sources to the marine interface (Sanders et al. 2022, Fig. 5). The Lena delta is a source principally of organic nitrogen to the Laptev Sea, as shown by high TDN, which vastly changes the N:P ratios in this region. However, the high N* and TDN* quickly decrease when higher salinities are reached. Excess nitrogen is preferentially removed compared to phosphorus when salinities increase from 5 to 28 psu on the shelf: TDN* decreases by 8.67 ± 7.90 μM and N* decreases by 7.43 ± 2.14 µM. As N* plots primarily below the mixing line in Fig. 5, nitrate is actively removed by N-cycling processes, returning to marine ratios of < 0 μM, where nitrogen becomes more limiting than phosphate to phytoplankton (Fig. 5).

Fluxes of nitrate & TDN in Arctic rivers vary strongly seasonally & spatially. During spring in the Lena delta, shallower sub-surface flows have higher nitrate:TDN ratios than during deeper, late summer fluxes, where groundwater origins are more important (Holmes et al. 2012; Connolly et al. 2020). This gradient is observed in our results: in spring, nitrate represents > 40% of TDN (nitrate = 10.10 ± 1.42 μM, TDN = 23.79 ± 3 μM). This drastically reduces in summer to < 3% (nitrate = 0.38 ± 0.42 μM, TDN = 13.47 ± 1.37 μM).

## Discussion

### Seasonal variability in surface macronutrients across the Barents Sea Opening

Our results highlight a seasonal change in the N:P stoichiometry in surface waters across the BSO, which is reset to the stoichiometry of AW in winter months. In the ice-free region of the southern Barents Sea, nutrient concentrations from the M/S Norbjørn transect are fully replenished, but do not reach maximum concentrations in surface waters until late winter (Fig. 2). The samples most influenced by freshwater output across the BSO (pink symbols in Fig. 2) appear to tie more closely with the salinity and nitrate dynamics of the ArW (blue line in Fig. 2), demonstrating that salinity stratification can play a large role in determining nutrient availability in surface waters. During summer months, nitrate is consumed faster than phosphate creating more N-limited conditions to phytoplankton. Although nitrate is more limiting than phosphate the non-zero nitrate concentrations observed in summer in the southern Barents Sea may alleviate nitrogen from being the sole limiting nutrient to primary production.

Recent nutrient limitation experiments in the Fram Strait have suggested that primary production in the AW is co-limited by the availability of nitrate, iron and silicate (Krause et al. 2019; Krisch et al. 2020). Our results show silicate concentrations decreasing below 1 mM in summer months at the Barents Sea Opening, suggesting diatom limiting conditions (Fig. 2g). As AW increases its areal extent, diatom limitation and blooms of non-silicifying species, such as *Emiliana huxleyi* and *Phaeocystis* spp., are becoming more prevalent (Neukermans et al. 2018; Orkney et al. 2020; Oziel et al. 2020). Diatoms account for much of polar primary production and carbon drawdown (Krause et al. 2019), and also have a high affinity for nitrate uptake (Glibert et al. 2016). Thus, any decrease in silicate availability via changes to circulation may influence the seasonal nutrient dynamics and the supply of organic matter to sediments and food to higher trophic levels (Vernet et al. 2017). Phytoplankton species shifts towards smaller, non-silicifying flagellates reduce the community level ability to consume nitrate compared to regenerated N forms (e.g. ammonium, urea, amino acids), with the potential to reduce net biological CO_2_ uptake and organic matter export (Reigstad et al. 2002). Whilst nitrate uptake is the primary source of phytoplankton N nutrition during the spring bloom, its importance diminishes later in the growing season, when regenerated N uptake becomes more important and can become dominant in mid-late summer if nitrate is limiting (Reigstad et al. 2002; Garneau et al. 2007). Regenerated N forms are also preferred by smaller non-diatom phytoplankton that tend to occur later in the seasonal succession of the phytoplankton community (Kristiansen et al. 1994; Signorini and McClain 2009).

### Supply and circulation of Atlantic Water

Recent warming of AW has been driven by a combination of both a local response to increasing air temperatures and reduced heat loss (Furevik 2001; Karcher et al. 2005), and a greater proportion of subtropical water being transported into the Nordic Seas (Hatun et al. 2005). Carried by the boundary current in the Eurasian Basin, this warming signal is propagating northwards into the Arctic basin and increasing heat fluxes to overlying water. This weakens the halocline, increases winter ventilation of the ocean interior and accelerates sea ice decline (Polyakov et al. 2020). These changing physical processes, combined with increasing primary production (Lewis et al. 2020), may be altering the nutrient inventory of AW (Fig. 5).

**Fig. 6:**
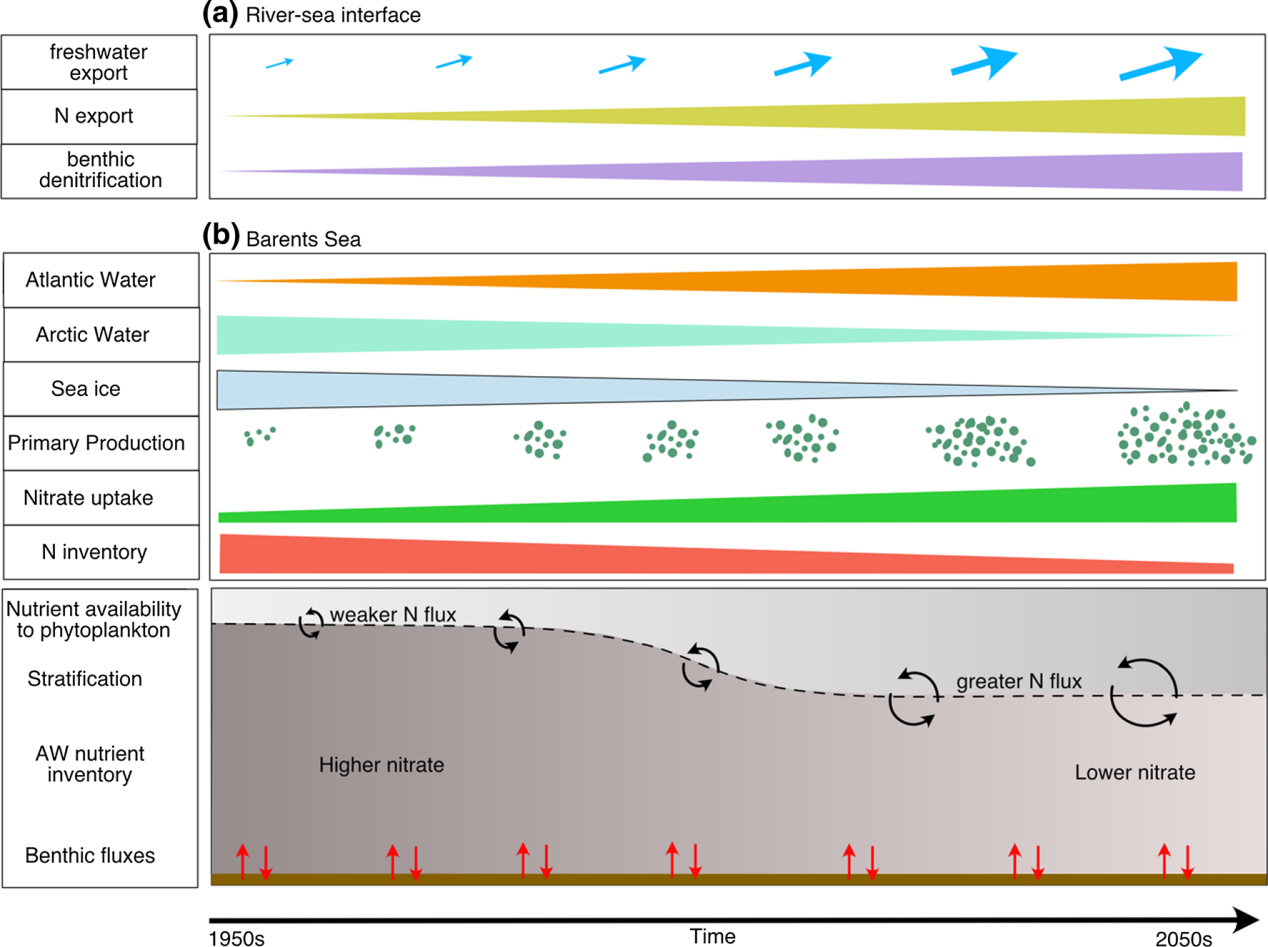
Observed and suggested changes to the Eurasian Arctic over time. The increases in primary production over time are combined with an increasing areal extent of AW, a decrease in ArW, loss of sea ice and weakening stratification. Our results suggest that these changes have led to an enhanced nitrate flux to the surface, but a higher uptake of nitrate by phytoplankton and higher primary production, therefore a decrease in summertime nutrients

Our decadal trends demonstrate decreases in the upper ocean nitrate and phosphate inventory in the Barents Sea, particularly in the upper 100 m (Fig. 3d). We suggest that although weaker stratification may be increasing the nutrient supply to surface waters through enhanced vertical nutrient fluxes (Randelhoff et al. 2016), increased primary production is leading to an overall loss in nutrients in the upper ocean in summer months. This suggests that the changes to the nutrient inventory are largely driven by primary production and changes to ventilation (Fig. 6). Recent findings of increased production in the Barents and Norwegian Seas suggest that this signal may either be locally or regionally driven (Lewis et al. 2020). Our results demonstrate that the annual average nutrient concentrations of deeper AW may also be decreasing, but to a lesser extent, which suggests that these uptake-driven changes are having less of an effect on the deeper AW because of winter convection and nutrient regeneration. These findings contrast with recent work in the central Arctic basin (Duarte et al. 2021), where no trend in AW was noted. We have used a different geographical range, temporal period, and sampling method all of which may explain these differences.

We also identify an increase in N* in the AW over time. This finding has also been identified through modelling work, which has suggested the enhancement of atmospheric deposition in the subtropical gyre as the mechanism driving this change (Buchanan et al. 2022). This has important implications for the Arctic, as the changing stoichiometry may partially alleviate N limitation in this region.

Overall, these temporal trends suggest that the upper ocean nutrient inventory of the Barents Sea is decreasing because of localised nutrient uptake and enhanced primary production. The different trends over the water column suggest that weakened stratification has led to a redistribution of nutrients, where there has been enhanced nitrate fluxes to the upper euphotic zone which may be sustaining enhanced primary production alongside increased light availability (Fig. 6).

### Arctic and Atlantic Water changes and the implications of sea ice loss

Comparison of seasonal nutrient dynamics between an ice-influenced, ArW-dominated site and an ice-free AW-dominated site revealed that in the latter, there was a greater supply of nitrate both before and during the spring/summer growing season, and nitrate drawdown occurred more slowly over a longer period of time (Henley et al. 2020). This finding corroborates our temporal trends, suggesting that the weakened stratification associated with Atlantification is increasing nitrate availability in surface waters of the Barents Sea. Nitrate is also resupplied more rapidly in autumn under ice-free, more Atlantic-

like conditions (Henley et al. 2020). These findings suggest a potential positive feedback whereby reduced sea ice cover, which is driven in part by the warming associated with increased prevalence of AW, enables greater convection, energetic tidal and wind-induced mixing of AW into surface waters, with further implications for warming and nutrient supply (Polyakov et al. 2020). In contrast, more extensive and longer-lasting ice cover is closely linked to stronger stratification, which slows down nutrient resupply by restricting vertical mixing (Randelhoff et al. 2015, Fig. 6). The relative importance of nitrate-based and regenerated production is strongly influenced by spatial and temporal variability in vertical mixing and nitrate supply, linked to variation in wind, tidal and topographic forcing (Randelhoff et al. 2015), and has significant consequences for biological CO_2_ uptake and organic matter export (Reigstad et al. 2002).

In addition to the impact of sea ice losses on pelagic production and nutrient uptake, primary production by sympagic (ice-associated) algae within or attached to the sea ice is even more susceptible to ongoing losses of the sea ice habitat. Ice algal primary production accounts for 2–10% of annual primary production in Arctic waters (Arrigo et al. 2017 and references therein), which presumably will diminish substantially as sea ice losses continue. However, it is expected that this sympagic primary production will be replaced by pelagic primary production, leading to overall increases in production (Arrigo and van Dijken 2015).

As well as the influence of sympagic primary production, sea ice processes also influence upper ocean biogeochemistry through enhanced nutrient (re)cycling within the semi-closed sea ice matrix by the sympagic microbial community (Meiners and Michel 2017 and references therein). This is particularly the case in spring and summer, and leads to a greater proportional contribution of regenerated N in the sea ice nutrient pool, which then influences upper ocean nutrient dynamics through ice-ocean exchanges. These exchanges and their impacts on upper ocean biogeochemistry will also be modified by ongoing sea ice losses, attendant changes in sea ice properties and the strengthening of upper ocean currents and shear linked with weakening stratification (Polyakov et al. 2020), and need to be considered in future projections of Arctic Ocean primary production and biogeochemical cycling.

### Benthic/pelagic coupling and loss of N via denitrification

In shallow seas, which comprise half of the Arctic Ocean, benthic processes can exert a strong influence on water column biogeochemistry and may drive future changes in the pelagic nutrient inventory. Close coupling of nitrification and denitrification in Arctic shelf sediments (McTigue et al. 2016) suggests that a significant proportion of fixed N lost through denitrification is derived from organic matter (OM) from the overlying water column. As such, changes in OM supply to sediments can drive significant changes in denitrification rates and therefore pelagic nitrate and N* inventories. Recent work has identified the importance of OM quality, as well as quantity, in modifying benthic nutrient recycling and fluxes (Freitas et al. 2020). In particular, the delivery of fresh OM with low C:N ratios (i.e. N-rich) to the sediments favours the recycling of nutrients back into the water column, thus reducing the loss of N from sediments through denitrification, compared to N-poor degraded material with high C:N ratios which may favour denitrification (Albert et al. 2021).

We hypothesise that under more Atlantic-like conditions there would be a greater delivery of OM to sediments and higher rates of benthic OM remineralisation. This could lead to increased nitrate and phosphate fluxes and increased sedimentary denitrification at AW-dominated stations. In the data presented within this study we find no statistically significant differences in benthic flux estimates between the AW and ArW regimes.

In the western Arctic, benthic denitrification rates are high, and with increases in primary production, denitrification rates are predicted to increase with enhanced OM supply to sediments (Chang and Devol 2009). In the Barents Sea, there is no significant change in the pelagic N:P inventory of imported and exported nutrients, suggesting that benthic denitrification may have a minimal impact on the fixed N inventory compared to western Arctic shelves (Tuerena et al. 2021b; Fig. 6). If the increase in primary production driven by weakened stratification and sea ice losses continue and translate to an increase in organic matter export and delivery to the seafloor, this could ultimately increase benthic nutrient recycling and fluxes back into the water column, as well as enhancing fixed N loss via denitrification. However, our results suggest that this is not likely to have a major influence on the shelf fixed N budget, as even with recent increases in Barents Sea primary production, denitrification is only a minor component of the N inventory and not significantly different between AW and ArW sites (Fig. 6).

The contrasting findings between the Barents Sea and western Arctic shelves may result from a number of factors. The Pacific inflow supplies the much shallower Chukchi and Beaufort shelves (< 60 m), with higher concentrations of macronutrients. In contrast, the Atlantic inflow to the Barents Sea provides lower concentrations of macronutrients to a deeper shelf (> 100 m). Substantially higher organic matter supply to the sediments of the western Arctic shelves would thus explain the much greater degree of benthic denitrification than we observe in the Barents Sea (Fig. 4; Chang and Devol 2009). Future changes in the loss of N through benthic denitrification in the Barents Sea will depend on changes in the quality and quantity of OM supplied to the sediments. However, at present, even with the increases in primary production that have been recorded since the 1990s, N loss is a minor term in the Barents Sea fixed N budget. As such, we suggest that benthic denitrification is unlikely to have a significant impact on the pelagic nutrient budget in future years (Fig. 6).

### Riverine inputs

The Arctic Ocean holds less than 2% of the ocean’s volume yet receives approximately 10% of total riverine fluxes. As such riverine export can influence the biogeochemistry of the coastal Arctic and also the pan-Arctic transport of properties via the Transpolar Drift (TPD). Terrestrial sources of N and P to the Arctic Ocean via rivers can significantly alter the coastal nutrient budget via organic and inorganic nutrient sources (Terhaar et al. 2021), but this freshwater also stratifies the upper ocean and restricts the renewal of nitrate from underlying waters (Tremblay and Gagnon 2009). Benthic denitrification may also rapidly remove excess N from in the vicinity of river mouths, exacerbating N limitation (Chang and Devol 2009).

Our results demonstrate a delivery of inorganic and organic nutrients with high N:P ratios to the coastal zone, however the N delivered is quickly removed from the water column at low salinities (Sanders et al. 2022, Fig. 5). On the Laptev Sea shelf, 62–76% of dissolved organic nitrogen (DON) released from the Lena river is removed within a couple of months (Thibodeau et al. 2017) and the importance of benthic denitrification has been identified in depleting N concentrations relative to P in the deeper waters of the continental slope (Bauch et al. 2011). These findings are confirmed in the bottom waters of the strongly stratified nearshore Laptev Sea, where N is rapidly depleted, and the sediment appears to be a source of phosphorus to the water column (Sanders et al. 2022). This aligns with estimates that riverine delivery of organic and inorganic N only has a minor (< 15%) contribution to Arctic shelf export production as 70% of terrestrial N is removed before reaching the marine endmember (Letscher et al. 2013), and a ninefold increase of riverine nitrate supply would be required to overcome nitrate-limitation in primary production on Arctic shelves (Le Fouest et al. 2013).

These seasonal trends, while observed in the Ob, Yenisey and Lena rivers, do not apply to other major Arctic rivers (Holmes et al. 2012). Thus, the trajectory of change of nutrient pathways with future warming of Arctic rivers may depend on individual river characteristics such as permafrost coverage, type and extent of vegetation, and glacial influence (Holmes et al. 2012). As the Lena river has 77% coverage of continuous permafrost (Holmes et al. 2013), permafrost thaw may enhance N (particularly DON) and P export to the Arctic Ocean, likely enhancing N availability at the coastal margin. Nevertheless, a 2–3 year residence time over the eastern shelves follows from the fast inflow of AW over the shelves, before export through the TPD (Karcher and Oberhuber 2002). This allows for the strong DON cycling to nitrate and benthic denitrification observed over the shelf to further modify river outputs over longer timescales, transforming N:P ratios closer to marine values in the TPD despite riverine seasonality, and finally, dampening the impact of excess N on Arctic-wide budgets. The capacity for East Siberian shelves to remove additional nitrogen sources from increased riverine fluxes over these long timescales remains undocumented.

In summary, Arctic riverine fluxes will increase with climate change (Rawlins et al. 2010), likely increasing DON & nitrate fluxes to Arctic shelves (Frey et al. 2007), and in turn increasing primary productivity around river deltas (Terhaar et al. 2021). Significant changes in N:P export at the pan-Arctic scale are however unlikely due to vigorous cycling of nitrogen on Arctic shelves. The large discrepancies in recent estimates of riverine N contribution to primary production (Le Fouest et al. 2013; Letscher et al. 2013; Terhaar et al. 2021) highlight the importance of benthic denitrification on Arctic shelves when considering Arctic-wide nutrient budgets, as well as the uncertainty associated with it.

## Societal and policy implications

Primary productivity underpins the entire Arctic ecosystem, yet we still lack a complete understanding of how this productivity is sustained through nutrient delivery (Table 2, Lewis et al. 2020). This dearth of knowledge stunts our ability to project how Arctic ecosystems will respond in the future as climate change enhances Arctic productivity (Vancoppenolle et al. 2013). Output from numerical models (Buchanan et al. 2022; Terhaar et al. 2021) alongside investment in sustained observations (Henley et al. 2020) or collation of data sets (Codispoti et al. 2013; Le Fouest et al. 2013) has the potential to address this shortcoming. However, key knowledge gaps remain regarding important aspects of Arctic nutrient dynamics, including (a) the impact of decreasing AW nutrient content on future primary production, (b) whether weakening stratification will continue with future projected warming, and (c) the role of increasing primary production and increased riverine N flux on benthic denitrification in the eastern Arctic Ocean (Table 2; Fig. 6).

**Table 2:** Processes influencing nutrient concentrations in the contemporary and future Eurasian Arctic Ocean as well as the level of confidence or evidence

Process	Current effect on nitrate	Level of confidence or evidence	Future prediction
Sea ice loss	Increased primary production drives **increased nitrate demand**	High: strong evidence for increase in primary production^a^ Medium: sparse observational evidence for increase in nitrate demand, but supported by model output^2^	Primary production is predicted to increase, thus further increasing nitrate demand^b,c^
Retraction of the subpolar gyre	Enhanced contribution of subtropical gyre water to Atlantic Water **decreases nitrate**	Low-medium: lack of decadal scale observational evidence on nutrients^d^	Decrease in total N supply to Eurasian Arctic via Atlantic Water^b^
Decreased stratification	Increased mixing **increases nitrate in surface waters**	High: Direct observations indicate reduced stratification, with implications for nitrate supply (medium)^e^	Short term: Increase in salinity and decline in sea ice expected to continue^c^, further weakening stratification, with potential for increased nitrate supply to surface waters Long term: Enhanced thermal stratification in upper water column may reduce nitrate resupply in winter months^c^
River inputs	Increased riverine discharge with potential for increased DON supply from permafrost thaw, but **low input of nitrate** to shelf seas due to efficient removal on shelf via denitrification	High: long-term evidence of change in riverine discharge and low nitrate delivery^f^ Low: increase in DON discharge from thawing permafrost^g, h^ Low: evidence for denitrification in Laptev/Kara seas^i^	Increase in riverine discharge, further increases in DON outflow but increases in N removal on shelf via denitrification
Sedimentary denitrification	Low rates in the Barents Sea cause **negligible contribution to nitrate removal**	Low: sparse measurements of rates or total N loss through sedimentary denitrification (*this study*)	Potential (slight) increase, although negligible due to ocean deoxygenation and increased primary production in the Barents Sea Potential for enhanced N loss in the Kara and Laptev seas

^a^Lewis et al. (2020), ^b^Buchanan et al. (2022), ^c^Vancoppenolle et al. (2013), ^d^Rey (2012), ^e^Lind et al. (2018), ^f^Rawlins et al. (2010), ^g^Frey et al. (2007), ^h^Holmes et al. (2013), ^i^Sanders et al. (2022)

The inextricable link between nutrient cycling, plankton at the base of the food web, and the higher trophic levels of fish, marine mammals and benthic organisms dictates that advances in our understanding of regional nutrient biogeochemistry are essential if we are to deliver sustainable management of Arctic marine resources now and into the future. These organisms support high-value commercial fisheries as well as the livelihoods and food security of Arctic communities, such that better quantification and more accurate projections of nutrient dynamics have the potential to inform local, national and intergovernmental decision-making around Arctic marine policy and management. As well as feeding into national and Arctic-wide governance frameworks (e.g. the Protection of the Arctic Marine Environment (PAME) working group of the Arctic Council), these advances will contribute more generally to the UN Sustainable Development Goals (2015) and the UN Decade of Ocean Science for Sustainable Development (2021–2030) in the Arctic context. New insight will allow scientists to disentangle the climate-driven, bottom-up drivers of the ecosystem from commercial fishing alongside natural variability.

## Supplementary Information

Below is the link to the electronic supplementary material.

Supplementary file1 (PDF 84 kb)
