# Supplementary material for: Nutrient pathways and their susceptibility to past and future change in the Eurasian Arctic Ocean
Source: Ambio. 2021 Dec 16;51(2):355–69. doi: 10.1007/s13280-021-01673-0 (PMC8692559; doi:10.1007/s13280-021-01673-0)
Supplement: Supplementary file 1 — Supplementary file1 (PDF 84 kb) [file 13280_2021_1673_MOESM1_ESM.pdf]

Supplementary Information for:

Nutrient pathways and their susceptibility to past and future change in the Eurasian Arctic Ocean

Temporal trends

Data used for temporal trends were taken within the geographical range of 70-80°N and 0-50°E. To overcome sampling variability, we only used summer nutrient measurements to look at changes in the upper water column (June-September). We also only used samples with salinities >34.6, this includes AW and ArW but reduces the effects of dilution on the nutrient concentrations and trends.

Table S1. 0-25m

**Dataset**	**Data source or DOI**	**Time range**	**Data points (NO3)**	**Data points (PO4)**	**Data points (N*)**
Codispoti2013	10.1016/j.pocean.2012.11.006	1990-2002	148	431	148
GIPY11	Geotraces IDP2017	2007	7	7	7
JR271	BODC	2012	13	13	13
PS94	BODC	2015	6	6	6
CAO - JR16006	doi:10.5285/b4c1537e-c729-6463-e053-6c86abc0c7de	2017	14	14	14
CAO - JR17006	10.5285/b62f2d5d-1f3f-0c2c-e053-6c86abc0265d	2018	12	3	3
CAO - JR17007	10.5285/b62f2d5d-1f40-0c2c-e053-6c86abc0265d	2018	3		
Kattner2011 PS68	Pangaea 10.1594/PANGAEA.761684	2005	51	53	46

Table S2. 0-100m

**Dataset**	**Data source or DOI**	**Time range**	**Data points (NO3)**	**Data points (PO4)**	**Data points (N*)**
Codispoti2013	10.1016/j.pocean.2012.11.006	1990-2002	168	514	168
GIPY11	Geotraces IDP2017	2007	9	9	9
JR271	BODC	2012	12	6	6
PS94	BODC	2015	8	8	8
CAO - JR16006	doi:10.5285/b4c1537e-c729-6463-e053-6c86abc0c7de	2017	20	16	16
CAO - JR17006	10.5285/b62f2d5d-1f3f-0c2c-e053-6c86abc0265d	2018	16		
CAO - JR17007	10.5285/b62f2d5d-1f40-0c2c-e053-6c86abc0265d	2018	7	3	3
Kattner2011 PS68	Pangaea 10.1594/PANGAEA.761684	2005	58	60	

Table S3. 200-300m

**Dataset**	**Data source or DOI**	**Time range**	**Data points (NO3)**	**Data points (PO4)**	**Data points (N*)**

Codispoti2013	10.1016/j.pocean.2012.11.006	1990-2002	149	149	149
JR271	BODC	2012	9	9	9
PS94	BODC	2015	4	4	4
CAO - JR16006	doi:10.5285/b4c1537e-c729-6463-e053-6c86abc0c7de	2017	7	7	7
CAO - JR17005	10.5285/b61d58df-b8e8-11c4-e053-6c86abc0246c	2018	2	2	2
CAO - JR17006	10.5285/b62f2d5d-1f3f-0c2c-e053-6c86abc0265d	2018	6	6	6
CAO - JR17007	10.5285/b62f2d5d-1f40-0c2c-e053-6c86abc0265d	2018	2	2	2
Kattner2011 PS68	Pangaea 10.1594/PANGAEA.761684	2005	45	45	45
